# Challenges in diagnosis of central nervous system infections

**DOI:** 10.1055/s-0044-1779027

**Published:** 2024-01-29

**Authors:** Avindra Nath, Bridgette Jeanne Billioux

**Affiliations:** 1 National Institutes of Health, National Institute of Neurological Disorders and Stroke, Global Neurology, Section of Infections of the Nervous System, Bethesda, Maryland, United States.; 2 National Institutes of Health, National Institute of Neurological Disorders and Stroke, Global Neurology, Bethesda, Maryland, United States.

Infections of the nervous system have devastating long-term effects. Early diagnosis is key to preventing permanent damage. Yet establishing the correct diagnosis in a short period of time poses many challenges. In the manuscript, Cortes et al., analyzed 496 CSF samples by a commercially available nested PCR assay, BioFire FilmArray. 1 The meningoencephalitis panel is designed to detect 14 pathogens. These include six bacteria (E. Coli, H influenza, Listeria monocytogenes, Neisseria meningitidis, Streptococcus agalactiae and Streptococcus pneumoniae), seven viruses (Herpes simplex 1 and 2, Human Herpesvirus 6, Cytomegalovirus, Varicella Zoster virus, Parechovirus and Enterovirus) and one fungus (Cryptococcus). The instrument is fully automated and self-contained as a single unit and uses only 200 ul of CSF. 2 Hence it can be easily used in most settings. Despite the limited number of pathogens in this assay, the authors found 88 samples that tested positive. Interestingly, enterovirus was found in nearly half of the samples. However, this approach has several limitations. The authors discovered a high false positive rate. This was particularly evident for the detection of bacteria since it could not be confirmed by repeat testing or by microbial cultures. Nested PCR is a highly sensitive assay, hence any contamination during the process of collection of the CSF can result in a false positive reading. There was no confirmatory test done for the viral pathogens, hence the accuracy of detection remains uncertain. The authors also do not report the clinical manifestations or clinical course of these patients to know the clinical significance of the findings.

The assay covers only 14 pathogens. Many common causes of meningoencephalitis such as the arboviruses are not included in the panel. The results are reported as positive or negative; hence, the amount of pathogens is not quantified. It is important to consider other limitations of this assay. Human Herpesvirus 6 (HHV-6) can become reactivated in many inflammatory conditions, and the detection of this virus does not necessarily indicate that it is pathogenic; in this study, three samples were positive for this virus. PCR is also not the most sensitive assay for the detection of fungi or cryptococcus, with antigen detection assays considered to be much more sensitive. In this study, three samples were positive for Cryptococcus. Listeria is an intracellular pathogen and may not leak into the CSF unless the brain abscess ruptures into the CSF space. 3 None of the samples here tested positive for Listeria. Importantly, no pathogen was detected in 408 samples. If the clinical course of these patients was that of a CNS infection, then complete reliance on this assay would be problematic. Other techniques such as next-generation sequencing 4 and methods used to concentrate the RNA by liquid hybridization followed by next-generation sequencing 5 should be considered for the detection of infectious agents, particularly where a viral infection is being considered. Cost is the major limiting factor for these technologies, particularly in resource-limited settings; however, early diagnosis can result in substantial savings in health care costs. While the Biofire Meningitis/Encephalitis panel does provide testing for several pathogens which may be helpful in the diagnosis of CNS infections, particularly where there may be a lack of skilled laboratory or PCR technicians, it also may be costly in settings with limited resources (in addition to the issues noted above). A cost-benefit analysis of such investigations would be the necessary first step in this process.
